# Abnormal retinal development associated with FRMD7 mutations

**DOI:** 10.1093/hmg/ddu122

**Published:** 2014-03-31

**Authors:** Mervyn G. Thomas, Moira Crosier, Susan Lindsay, Anil Kumar, Masasuke Araki, Bart P. Leroy, Rebecca J. McLean, Viral Sheth, Gail Maconachie, Shery Thomas, Anthony T. Moore, Irene Gottlob

**Affiliations:** 1 Ophthalmology Group, School of Medicine, University of Leicester, RKCSB, PO Box 65, Leicester LE2 7LX, UK; 2 MRC-Wellcome Trust Human Developmental Biology Resource (Newcastle), Institute of Human Genetics, Newcastle University, International Centre for Life, Newcastle upon Tyne NE1 3BZ, UK; 3 Department of Biological Sciences, Developmental Neurobiology Laboratory, Nara Women's University, Nara 630-8506, Japan; 4 Department of Ophthalmology and Centre for Medical Genetics, Ghent University and Ghent University Hospital, Ghent 9000, Belgium; 5 Department of Ophthalmology, Nottingham University Hospital NHS Trust, Nottingham, UK; 6 UCL Institute of Ophthalmology, London EC1V 9EL, UK

## Abstract

Idiopathic infantile nystagmus (IIN) is a genetically heterogeneous disorder, often associated with *FRMD7* mutations. As the appearance of the retina is reported to be normal based on conventional fundus photography, IIN is postulated to arise from abnormal cortical development. To determine whether the afferent visual system is involved in *FRMD7* mutations, we performed *in situ* hybridization studies in human embryonic and fetal stages (35 days post-ovulation to 9 weeks post-conception). We show a dynamic retinal expression pattern of *FRMD7* during development. We observe expression within the outer neuroblastic layer, then in the inner neuroblastic layer and at 9 weeks post-conception a bilaminar expression pattern. Expression was also noted within the developing optic stalk and optic disk. We identified a large cohort of IIN patients (*n* = 100), and performed sequence analysis which revealed 45 patients with *FRMD7* mutations. Patients with *FRMD7* mutations underwent detailed retinal imaging studies using ultrahigh-resolution optical coherence tomography. The tomograms were compared with a control cohort (*n* = 60). The foveal pit was significantly shallower in *FRMD7* patients (*P* < 0.0001). The optic nerve head morphology was abnormal with significantly decreased optic disk area, retinal nerve fiber layer thickness, cup area and cup depth in *FRMD7* patients (*P* < 0.0001). This study shows for the first time that abnormal afferent system development is associated with *FRMD7* mutations and could be an important etiological factor in the development of nystagmus.

## INTRODUCTION

Infantile nystagmus is characterized by involuntary to and fro movements of the eyes, which is present at birth or manifesting within the first few months of life. Nystagmus has an estimated prevalence of 2.4 in 1000 (1) and is associated with significant negative social stigma and poor visual function scores (2,3). The pathophysiology of this disorder is unclear, although numerous hypotheses have been put forward. Previous animal models for infantile nystagmus have suggested that axonal misrouting at the level of the chiasm could be a common mechanism (4). Certainly in patients with albinism and achiasma, both associated with infantile nystagmus, misrouting of retinal ganglion cell axons within the retinofugal pathway at the level of the chiasm is observed. However, in many other forms of infantile nystagmus [e.g. aniridia, achromatopsia, idiopathic infantile nystagmus (IIN)] visually evoked potentials show interhemispherical symmetry, suggestive of normal decussation of retinal ganglion cell axons (5). The fact that most forms of infantile nystagmus arise due to mutations of genes expressed within the developing retina would argue in favor of an afferent abnormality. Moreover, abnormal retinal phenotypes have been described in most of these disorders (6–8). However, in IIN, other than reduced visual acuity and an abnormal optokinetic response (9,10), no overt ocular abnormality has been described. This has led to a number of mathematical models suggesting that infantile nystagmus arises due to the instability of the neural integrator (11,12) or the smooth pursuit system (13), rather than an afferent defect.

Mutations in *FRMD7* are a major cause of IIN (14). The *FRMD7* gene is located at Xq26.2. In male subjects with pathogenic *FRMD7* mutations, the disease is fully penetrant; however, in females with heterozygous mutations, the penetrance is ∼53% (9). *FRMD7* expression studies indicate anatomic pathways involved in the optokinetic reflex (9,10,14,15). However, detailed spatiotemporal expression within developing retina has not been characterized. FRMD7 promotes neurite elongation at the actin-rich growth cone ends through the modulation of actin cytoskeleton (16,17). *FRMD7* knockdown during neuronal differentiation alters neurite development, indicating a role in axonogenesis or dendritogenesis (16). Recently, it has been shown that CASK recruits FRMD7 to the plasma membrane to promote neurite outgrowth during development of the oculomotor neural network and disruption of this interaction results in nystagmus (17).

With the advent of optical coherence tomography (OCT), it is possible to visualize the retina (18) in much greater detail than conventional imaging techniques such as fundus photography. Previously, the use of OCT (time domain) in nystagmus was limited due to fixation instability, poor resolution and slow scanning speeds. The new generation spectral-domain OCTs are able to achieve faster scanning speeds and much higher resolution than the time domain instruments. This has enabled imaging in patients with infantile and acquired forms of nystagmus. Recent OCT studies in multiple sclerosis have suggested that this imaging modality has an important role in monitoring disease activity (19) and the retinal changes reflect global CNS processes (20). Similarly, OCT studies in infantile nystagmus have highlighted the spectrum of abnormal retinal phenotypes and its role in predicting visual acuity (7). We have recently shown that we can obtain reliable thickness measurements in patients with nystagmus using an ultrahigh-resolution OCT with very fast scanning speeds (21). During foveal pit formation (area of high acuity), the inner retinal layers are displaced centrifugally (away from the future foveal pit), while the cone photoreceptors migrate centripetally (toward the future foveal pit). As the cone photoreceptors migrate toward the fovea, they also undergo specialization, which involves lengthening of the outer segment. This allows increased packing of the cone photoreceptors with highest concentration at the fovea (22,23). OCT allows accurate documentation of the stages of arrested retinal development (7). The normal retinal laminar structure and foveal pit visualized using an ultrahigh-resolution OCT is shown in Supplementary Material, Figure S1.

To date, there have been no studies investigating systematically the retinal morphology in patients with IIN associated with *FRMD7* mutations. Studying the neural substrates involved in disease pathogenesis can point to potential therapeutic targets. Hence, we aimed to carefully investigate the afferent pathway, using high-resolution *in situ* hybridization techniques and retinal imaging in patients with *FRMD7* mutations.

## RESULTS

### FRMD7 expression

**Figure 1.**
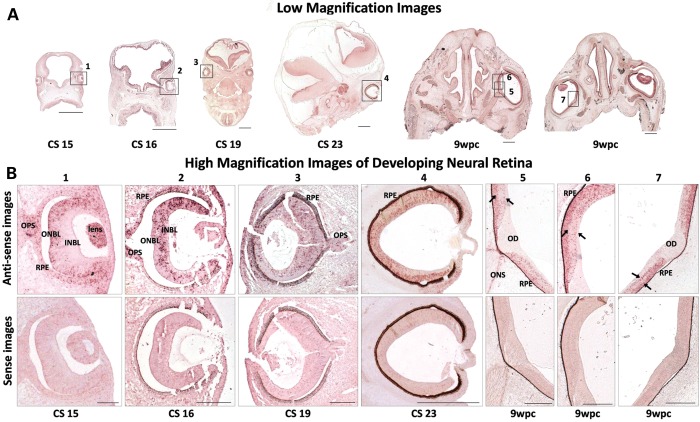
FRMD7 mRNA expression profile in developing human neural retina. (**A**) Low-magnification image of the embryos from CS 15, 16, 19, 23 and 9 weeks postconception (wpc). (**B**) Dynamic expression pattern; expression initially confined to the outer neuroblastic layer (ONBL) at CS15, subsequently expression seen within the inner neuroblastic layer (INBL). Bilaminar expression pattern at 9 wpc (arrows). Expression within the developing optic stalk (OPS) at CS15, CS16 and CS19. Expression restricted to the optic nerve sheath (ONS), absent in developing optic disk (OD) at 9 wpc. Peripheral neural retina is the last to differentiate and laminate, hence differential expression between central and peripheral neural retina, most evident at CS16 and CS19. Sense images shown below the antisense images; once pigmentation occurs, RPE appears as false positive for expression at CS16 onward. Low-magnification image scale bar: 500 μm high-magnification image scale bar: 200 μm.

We show a dynamic expression pattern of FRMD7 mRNA in the developing neural retina between Carnegie stages CS15–CS23 and 9 weeks postconception (wpc). Carnegie Stages 15–23 represent the embryological stages from 33 days post-conception (dpc) to 56 dpc. At CS15 (33 dpc) restricted *FRMD7* expression is seen within the outer neuroblastic layer (Fig. 1). Development of the retinal ventricular zone (VZ) is similar to the cortical VZ. Postmitotic cells from VZ migrate to the future ganglion cell and inner nuclear layers (24). These neurons are closely apposed to radial glia, their processes extending to the vitreal surface. At CS16 (37 dpc) *FRMD7* expression is seen within the inner neuroblastic layer. Differentiation of the retina begins at the optic disk, and then extends peripherally toward the rim. Thus, the staining pattern is different between central and peripheral retina at CS16 and CS19 (47 dpc). At CS23 (56 dpc) a bilaminar expression pattern emerges, which is evident by 9 wpc. There is *FRMD7* expression in the optic stalk at CS15, CS16 and CS19, while, at 9 wpc, expression is confined to the optic nerve sheath.

Based on our *FRMD7* expression results, we assessed retinal structure in *FRMD7* patients by measuring foveal pit depth and central macular and photoreceptor outer segment thickness. Since *FRMD7* regulates neurite outgrowth, we also measured retinal nerve fiber layer thickness, and optic disk area, a measure of optic nerve fiber count (25).

### Retinal phenotypes associated with FRMD7 mutations

**Figure 2.**
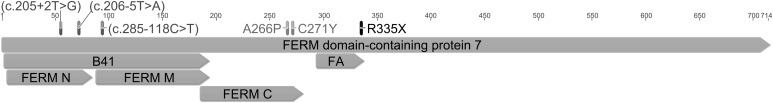
FRMD7 mutations associated with foveal hypoplasia. All mutations were predicted to disrupt the FERM domain or the FERM-adjacent (FA) domain. Splice variants are shown in brackets. The resulting amino acid variations for missense and nonsense mutations are shown.

OCT identified an obvious failure of inner retinal cells to migrate away from the foveal pit (foveal hypoplasia) in 12 of 45 patients (Supplementary, Material, Table S2). The incursion of inner retinal layers posterior to the foveola represents the hallmark of foveal hypoplasia. Foveal hypoplasia was associated with missense, splice and nonsense mutations (Fig. 2), without obvious genotype–phenotype correlations. The c.285-118C > T and c. 206-5T > A represent novel mutations. The c.285-118C > T mutation is predicted to activate a cryptic splice donor within intron 4 (26). The c. 206-5T > A mutation is predicted to result in obliteration of the splice acceptor site in intron 3. The splice variants c.205 + 2T > G has previously been described (14). The c.205 + 2T > G is within the conserved splice donor residues (position + 1 and +2) and is thus predicted to be pathological by classical exon skipping and nonsense-mediated decay (14). Furthermore, the translational products are likely to be subject to nonsense-mediated decay. The missense mutations resulting in amino acid variants A266P and C271Y are predicted to be pathological (10,14). The C271Y is predicted to destabilize the FRMD7 protein by the introduction of a larger amino acid within restricted areas of the protein, whereas A266P is predicted to disrupt a helical domain within the wild-type protein (10,14). The nonsense mutation resulting in the amino acid variant R335X is predicted to be pathological due to introduction of a premature stop codon resulting in a truncated protein (10,14). *In vitro* studies have shown nuclear localization associated with R335X (17,27).

**Figure 3.**
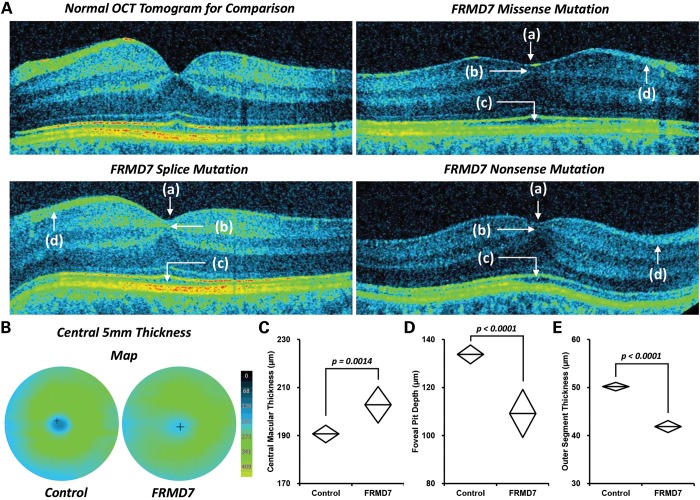
Foveal hypoplasia with FRMD7 mutations. (**A**) Arrested retinal development with FRMD7 mutations shown by shallow foveal pit (a), failure of inner retinal cell migration (b), failure of cone photoreceptor specialization (c) and smaller retinal nerve fiber layer (d). (**B**) 3D thickness maps showing rudimentary foveal pit compared with controls. Central macular thickness (**C**), foveal pit depth (**D**) and outer segment thickness (**E**) were significantly different compared with controls.

The foveal pit depth was significantly decreased in the FRMD7 patients in comparison with the controls (mean difference = 24.6 µm, *P* < 0.0001). The central macular thickness was significantly increased in patients with *FRMD7* mutations in comparison with controls (mean difference = 12.1 µm, *P* = 0.0014). A shallow foveal pit and increased central macular thickness are consistent with foveal hypoplasia (Fig. 3). We did not identify any cases of fovea plana (i.e. anatomic lack of foveal pit). None of the control subjects had foveal hypoplasia. In order to assess the maturity of the outer retina and degree of photoreceptor specialization, we assessed the cone outer segment length. We noted that the cone outer segment was significantly decreased in length in patients with *FRMD7* mutations when compared with controls (mean difference = 8.3 µm, *P* < 0.0001).

**Figure 4.**
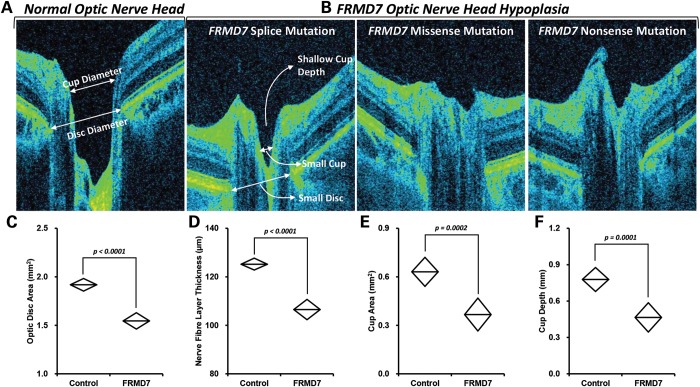
Optic nerve changes with FRMD7 mutations. (**A**) Normal optic nerve head shown for comparison with FRMD7 mutations (**B**). Optic disk area (**C**), nerve fiber layer thickness (**D**), cup area (**E**) and cup depth (**F**) were significantly different compared with controls.

Based on the FRMD7 expression within the optic nerve head in humans, we performed quantitative measurements in the patients with *FRMD7* mutations and compared it with the controls. e identified that the average peripapillary retinal nerve fiber layer thickness was significantly decreased in patients with *FRMD7* mutations when compared with controls (mean difference = 18.7 µm, *P* < 0.0001). Similarly, the optic disk area was significantly decreased in patients in comparison with controls (mean difference = 0.37 mm^2^, *P* < 0.0001). The optic nerve cup was shallower (mean difference = 0.31 mm, *P* = 0.0001) with decreased cup area (mean difference = 0.26 mm, *P* = 0.0002) in patients with *FRMD7* mutations (Fig. 4).

The mean visual acuity in the FRMD7 cohort was 0.20 LogMAR. There was no significant difference in visual acuity or OCT measurements between male and female patients (*P* > 0.05).

## DISCUSSION

This study shows for the first time that patients with IIN have retinal and optic nerve changes. This raises an interesting possibility that afferent defects during early development could underlie the development of childhood nystagmus. Foveal hypoplasia is typically associated with inherited developmental retinal disorders such as albinism, *PAX6*-related phenotypes and achromatopsia (7,28). Infantile nystagmus is a common feature of these conditions. During fovea formation, there is centrifugal displacement of inner retinal cells as the foveal pit deepens, and centripetal migration and specialization of cone photoreceptors (22,23). In patients with *FRMD7* mutations, impaired growth cone guidance (14,16) could lead to retinal neuron migratory defects such as foveal hypoplasia and developmental abnormalities of the optic nerve head. This is consistent with expression patterns we observed in the developing retina and optic nerve.

The predominant clinical features observed in patients with *FRMD7* mutations are reduced visual acuity and abnormalities of the optokinetic response (9,10,15). The novel finding of foveal hypoplasia could be the basis of these abnormalities previously described. Typically, patients with *FRMD7* mutations tend to have better visual acuity compared with patients with albinism (29). The foveal hypoplasia observed in patients with *FRMD7* mutations is much milder (i.e. patients with foveal hypoplasia had at least a rudimentary pit—Grade 1 foveal hypoplasia) in comparison with albinism, where the majority of patients had no foveal pit (Grade 3 foveal hypoplasia) (7). This could explain the better visual acuity previously reported in *FRMD7* groups than in albinism (29).

Previously, there have been no reports of retinal defects in patients with idiopathic nystagmus. This is likely due to the limitations associated with retinal imaging. The advent of spectral-domain optical coherence tomography has opened ultrahigh-resolution imaging at high speeds. In this study, we have used one of the highest resolution commercially available instruments (axial resolution, 3 µm) with the fastest scanning speed (52 000 A-scans/s). We have shown that, using this instrument, we are able to perform reproducible OCT measurements in patients with nystagmus (21).

Further studies will be required to investigate how *FRMD7* mutations lead to the morphological retinal changes described in this study. Further studies in an animal model would be the next logical step in understanding the pathogenesis of this disorder. The FRMD7 knockout mouse model would be suitable model for further investigating the optic nerve changes and nerve fiber layer changes described in this study. However, a drawback of the mouse model is the lack of a fovea, so comparisons with the human visual system would be difficult. Thus, it would not be possible to deduce how *FRMD7* mutations lead to foveal hypoplasia. There have been no detailed neuroimaging studies in patients with *FRMD7* mutations. Therefore, it is unclear whether abnormal retinal development in *FRMD7* patients would lead to abnormal cortical development. Further neuroimaging studies coupled with OCT studies in humans as well as histological studies in FRMD7 knockout mouse would be needed to assess how the afferent abnormalities might affect neural circuitry within the oculomotor system.

To date there are only two genes, *PAX6* and *SLC38A8*, mutations of which can result in isolated foveal hypoplasia (30–32). *PAX6* mutations have been reported to be a rare cause of isolated foveal hypoplasia (28). *PAX6* mutations can also be associated with aniridia, optic nerve hypoplasia and brain abnormalities (33,34). *SLC38A8* mutations are also associated with developmental delay and pervasive developmental disorder-like features (30). Recently, Perez *et al*. used a combination of linkage analysis and whole-exome sequencing to identify a mutation in *SLC38A8* that results in infantile nystagmus and isolated foveal hypoplasia (30). However, the mutation was only found in a community of Jewish Indian ancestry, thus representing a very rare autosomal recessive cause of foveal hypoplasia. Poulter *et al*. expanded the mutation and phenotypic spectrum associated with *SLC38A8*, showing that mutations were also associated with optic nerve decussation defects, anterior segment abnormalities, but no other characteristics of albinism such as pigmentary abnormalities, including iris transillumination defects (32). SLC38A8 is expressed within the retina and fetal brain (30,32). With the advent of next generation sequencing technologies, it will become easier to identify more genes associated with foveal hypoplasia, thus providing greater understanding of the genetic basis of foveal development. In patients with *FRMD7* mutations, there have been no reports of brain abnormalities or other ocular phenotypes to date. We did not screen for *PAX6* mutations, since clinically there were no features to suggest *PAX6*-related phenotype. Moreover, it is also unlikely that our patients had *PAX6* mutations, since, as we have shown, in patients with *PAX6* mutations the nystagmus form is very different, usually with a vertical component (31). None of the families had male-to-male inheritance to suggest autosomal dominant nystagmus, as in *PAX6*, and all families were compatible with X-linked inheritance, as occurs in *FRMD7* mutations.

Our study suggests that *FRMD7* mutations can present as isolated foveal hypoplasia. Therefore, patients presenting with infantile nystagmus and foveal hypoplasia in the absence of other ocular anomalies should be considered for screening of *FRMD7* mutations. The study raises an interesting possibility that early sensorimotor integration failure underlies IIN development. Prior to this study, there have been no structural abnormalities associated with IIN, and IIN was thought to arise from abnormal cortical development. The findings in this study suggest that arrested retinal development is an important etiological factor in the development of nystagmus. Whether this in turn affects cortical development would need further study.

## MATERIALS AND METHODS

### Tissue *in situ* hybridization

Spatiotemporal *FRMD7* retinal expression was investigated by *in situ* hybridization (10,35) on human embryonic and fetal tissue. Human embryos were obtained with appropriate maternal written consent and approval from the Newcastle and North Tyneside NHS Health Authority Joint Ethics Committee. HDBR is regulated by the UK Human Tissue Authority (HTA; www.hta.gov.uk) and operates in accordance with the relevant HTA Codes of Practice.

Samples were fixed overnight at 4°C in 0.1 m phosphate buffered saline (PBS) containing 4% paraformaldehyde (PFA; Sigma–Aldrich, Poole, UK). The embryos were classified into Carnegie stages (36,37).

ISH was performed as previously described (38) with some modifications. Paraffin sections were dewaxed and rehydrated before being incubated with proteinase K (20 μg/ml; Sigma–Aldrich) for 8 min at room temperature. Sections were fixed in 4% PFA/PBS for 20 min, washed in PBS, and treated with 0.1 m triethanolamine (Sigma–Aldrich, pH 8.0)/0.25% acetic anhydride (Sigma–Aldrich)/0.2% HCl for 10 min, dehydrated in ethanol and air-dried. DIG-labeled probes (300 ng) were used per 100 μl of DIG Easy Hyb mixture (Roche, Lewes, UK). Probe/Hyb mix (200 μl) was used per slide, covered with glass coverslips. Slides were incubated in a hybridization chamber overnight at 68°C, rinsed in 5× standard sodium citrate (SSC; pH 7.2) at 65°C to remove coverslips, followed by three washes at 50°C (2× SSC twice and 0.2× SSC once), followed by one wash with 0.2× SSC once at room temperature. After briefly rinsing in 0.1 m Tris (pH 7.6)/0.15 m NaCl (Buffer 1) and blocking with 10% fetal calf serum (Invitrogen)/Buffer 1 for 1 h at room temperature, sections were incubated with anti-DIG antibody (Roche; diluted 1: 1000 in 2% FCS/Buffer 1) overnight at 4°C. Sections were washed in Buffer 1 for 6 × 30 min. Detection of probes/anti-DIG antibody was achieved by addition of NBT/BCIP solution (Roche; 20 μl/ml) in 0.1 m Tris (pH 9.5)/0.1 m NaCl (Buffer 2). The color reaction was developed in the dark for several hours to overnight and terminated by rinsing slides in Buffer 2 and then distilled water. Sections were mounted in Aquamount. Comparison of staining between sense and antisense probes was carried out to ensure specificity. Human embryos ranging in age from Carnegie Stage 15 (35 days postovulation) to 9 weeks postconception were obtained from the MRC/Wellcome Trust funded Human Developmental Biology Resource at Newcastle University (HBDR, http://www.hdbr.org).

### Subjects

Patients with nystagmus were recruited from different sites: University Hospitals Leicester (UK), Moorfields Eye Hospital (UK) and Ghent University Hospital (Belgium). Patients underwent detailed ophthalmic examinations, eye movement recordings and electrodiagnostic tests. A diagnosis of IIN was obtained based on (i) normal electrodiagnostic tests (electroretinograms and visually evoked potentials based on ISCEV standards), (ii) onset of nystagmus (horizontal, conjugate oscillation of the eyes) within the first 6 months of life (iii) no iris transillumination defects on slit lamp biomicroscopy and (iv) normal color vision on Ishihara testing. Informed consent was obtained from all participants in accordance to the Declaration of Helsinki and all protocols were approved by the local ethics committee. We identified a total of 100 IIN patients based on these diagnostic criteria. All IIN patients underwent sequence analysis to identify *FRMD7* mutations (see Materials and Methods). We wanted to investigate the retinal morphology in a homogeneous population; therefore, we only included patients with *FRMD7* mutations. Forty-five patients with *FRMD7* mutations were recruited for subsequent high-resolution retinal phenotyping using optical coherence tomography.

The control cohort (*n* = 60; mean age = 35.0 years, SD = 13.8, range = 5–62 years) was age, race and gender matched to the FRMD7 cohort (*n* = 45; mean age = 34.7 years, SD = 17.4, range = 7–79 years). The control cohort consisted of 32 males and 28 females. The FRMD7 cohort consisted of 25 males and 20 females. Patients with *FRMD7* mutations had only small refractive errors within ±3 D. The inclusion criteria for controls were refractive errors within ±3 D. During the scan protocol, refractive error data were entered in SOCT before examination. The SOCT software performs a refractive compensation.

Each control subject underwent an ophthalmic examination to exclude significant ophthalmic pathologies. There was no history of retinopathy of prematurity or other ophthalmological or neurological pathology in either cohort.

### DNA sequencing and analysis

Primers were designed to amplify the coding exons and the intron–exon boundaries of the *FRMD7* gene (Accession ID: NG_012347.1) (Supplementary Material, Table S1). All the coding exons and splice junctions were Sanger sequenced bidirectionally in affected subjects. Mutation analysis software SeqMan Pro v11.2 (DNAStar, Madison, WI, USA) was used for base calling and alignment of the contigs. Base position + 1 corresponded to A of the translation initiation codon ATG (Genbank file: NM_194277.2). Intronic sequence changes were identified based on the FRMD7 genomic sequence (NG_012347.1) and amino acid changes were identified based on the reference protein sequence (NP_919253.1). Allelic variants were reported according to Human Genome Variation Society guidelines. Allelic variations were assessed against the sequence data from 300 male controls (without nystagmus) and dbSNP database (http://www.ncbi.nlm.nih.gov/SNP/; dbSNP Build ID: 137). Novel intronic variants were also assessed using the Alternative Splice Site Predictor (26). Disease causing mutations were identified based on variant segregation with the phenotype, absence of the variants in the control samples and control databases and predicted effects on protein structure. The variant data have been submitted to LOVD database (www.LOVD.nl/MR).

### Optical coherence tomography

Ultrahigh-resolution spectral-domain OCT (SOCT Copernicus HR; OPTOPOL Technology SA) was used to acquire tomograms from both eyes of the patients (*n* = 45) and controls (*n* = 60). We have previously described the acquisition and analysis methods used in patients with nystagmus (7,21). This OCT uses a superluminescent light emitting diode at a central wavelength of 855 nm. A 3D scan program (743 × 75, A × B) was used to capture the foveal and parafoveal regions. The scanning window covered a 7 × 7 mm retinal area centered at the fovea. For optic nerve head acquisition, the same scan parameters were used; however, the fixation spot was altered and the scan window was centered at the optic nerve head. The terminations of the retinal pigment epithelium (RPE) were used to determine the borders of the optic disk; an anterior offset of 150 µm from the RPE was used to determine the borders of the cup. The acquired images were analyzed using the SOCT software (version 4.1) and custom scripts in ImageJ software (39) (Rasband, W.S., ImageJ, U.S. National Institutes of Health, Bethesda, MD, USA, http://imagej.nih.gov/ij/, 1997–2013). We have previously reported the reproducibility of OCT acquisition and analysis in patients with nystagmus (21).

The effective axial and transverse resolutions obtained using this machine were ∼3 and 12 µm, respectively, with a scanning speed of 52 000 A-scans/s. The foveal and optic nerve head B-scans were segmented and analyzed for morphological abnormalities and thickness measurements. These included foveal pit depth, central macular thickness, outer segment thickness, peripapillary nerve fiber layer thickness, disk area, cup area and cup depth.

### Statistical analyses

A linear mixed model was used to assess whether there were significant differences between the control cohort and patients with *FRMD7* mutations for retinal thickness measurements (foveal pit depth, central macular thickness, outer segment thickness, nerve fiber layer thickness, optic disk area, optic cup area and cup depth). Age, gender and race were used as random effects factors. Statistical analyses were performed in IBM^®^ SPSS^®^ Statistics version 20. The diamond plots were used in figures to show differences in thickness measurements. They represent the 95% confidence intervals with the line bisecting the diamond representing the mean.

## SUPPLEMENTARY MATERIAL

Supplementary Material is available at *HMG* online.

*Conflict of Interest statement.* None declared.

## FUNDING

This study was supported by the Ulverscroft foundation and the Medical Research Council (MRC), London, UK (grant number: MR/J004189/1). M.G.T. was supported by the National Eye Research Centre studentship (Grant No: SCIAD 057). The human embryonic and fetal material was provided by the Joint MRC (grant #G0700089)/Wellcome Trust (grant #GR082557) Human Developmental Biology Resource (http://hdbr.org). B.P.L. is a senior clinical investigator of the Fund for Research Flanders, Belgium. Funding to pay the Open Access publication charges for this article was provided by RCUK.

## Supplementary Material

Supplementary Data
