# Supplementary material for: Abnormal retinal development associated with FRMD7 mutations
Source: Hum Mol Genet. 2014 Mar 31;23(15):4086–93. doi: 10.1093/hmg/ddu122 (PMC4082370; doi:10.1093/hmg/ddu122)
Supplement: Supplementary Data [file supp_23_15_4086__index.html]

Abnormal Retinal Development Associated with FRMD7 Mutations — Abnormal retinal development associated with FRMD7 mutations — Abnormal retinal development associated with FRMD7 mutations — Supplementary Data 

# Abnormal retinal development associated with FRMD7 mutations

## Supplementary Data

Supplementary Data

**Files in this Data Supplement:**

- Supplementary Table 2 - pdf file
- Supplementary Figure 1 - doc file
- Supplementary Table 1 - docx file
