# Supplementary material for: Abnormal retinal development associated with FRMD7 mutations
Source: Hum Mol Genet. 2014 Mar 31;23(15):4086–93. doi: 10.1093/hmg/ddu122 (PMC4082370; doi:10.1093/hmg/ddu122)
Supplement: Supplementary Data [file supp_ddu122_ddu122supp_table2.pdf]

Patient ID	Gender	Age	DNA variant	Aminoacid variant	Visual acuity (LogMAR)	Foveal Pit present	Nystagmus Present	Foveal Pit depth	Central Macular Thickness	OS	Continuation of Inner retinal layers posterior to foveola	Reference for variant
NYS597	M	11	c.205+2 T>G		0.18	Yes	Yes	128.7	197.0	48.70	No	1
NYS1337	M	46	c.205+2 T>G		0.18	Yes	Yes	117.0	200.9	37.05	No	1
NYS612	F	9	c.205+2 T>G		0.18	Yes	Yes	120.9	198.9	44.85	No	1
NYS374	F	16	c.205+2 T>G		0.30	Yes	Yes	60.5	253.5	39.00	Yes	1
NYS1766	M	41	c.252G>A	p.V84V	0.18	Yes	Yes	119.0	206.7	42.90	No	1
NYS970	F	15	c.796G>C	p.A266P	0.00	Yes	Yes	122.9	181.4	40.95	No	1, 2
NYS1485	F	14	c.796G>C	p.A266P	0.20	Yes	Yes	74.1	212.6	40.95	Yes	1, 2
NYS1469	M	50	c.796G>C	p.A266P	0.10	Yes	Yes	99.5	200.9	46.80	No	1, 2
NYS120	F	46	c.162+5 G>A		0.18	Yes	Yes	119.0	195.0	40.95	No	1
NYS565	M	22	c.162+5 G>A		0.18	Yes	Yes	128.7	206.7	46.80	No	1
NYS1078	M	44	c.1003C>T	p.R335X	0.18	Yes	Yes	126.8	197.0	48.75	No	1, 2, 3
NYS1177	M	48	c.1003C>T	p.R335X	0.20	Yes	Yes	109.2	187.2	44.85	No	1, 2, 3
NYS942	F	10	c.1003C>T	p.R335X	0.30	Yes	Yes	93.6	224.3	40.95	No	1, 2, 3
NYS617	F	50	c.812G>A	p.C271Y	0.40	Yes	Yes	52.7	257.4	40.95	Yes	1, 2
NYS1664	M	21	c.812G>A	p.C271Y	0.18	Yes	Yes	130.7	179.4	39.00	No	1, 2
NYS1585	F	51	c.812G>A	p.C271Y	0.60	Yes	Yes	52.7	237.9	35.10	Yes	1, 2
NYS245	M	71	c.812G>A	p.C271Y	0.40	Yes	Yes	81.9	214.5	35.10	Yes	1, 2
NYS673	F	28	c.812G>A	p.C271Y	0.10	Yes	Yes	113.1	195.0	46.80	No	1, 2
NYS1410	M	38	c.661A>G	p.N221D	0.00	Yes	Yes	113.1	197.0	42.90	No	1
NYS105	F	40	c.1019C>T	p.S340L	0.00	Yes	Yes	119.0	183.3	37.05	No	1, 2
NYS36	F	44	c.71G>A	p.G24E	-0.10	Yes	Yes	134.6	169.7	40.95	No	1
NYS1436	M	16	c.71G>A	p.G24E	0.20	Yes	Yes	130.7	171.6	37.05	No	1
NYS1734	M	56	c.1003C>T	p.R335X	0.30	Yes	Yes	27.3	230.1	35.00	Yes	1, 2, 3
NYS107	F	35	c.796G>C	p.A266P	0.18	Yes	Yes	44.9	239.9	35.10	Yes	1, 2
NYS1965	M	34	c.58-1G>A		0.16	Yes	Yes	150.2	177.5	44.85	No	2
NYS1561	F	54	c.983A>G	p.Y328C	0.18	Yes	Yes	154.1	165.8	40.95	No	Novel
NYS1537	F	30	c.983A>G	p.Y328C	0.10	Yes	Yes	113.1	187.2	44.85	No	Novel
NYS131	M	30	c.47T>C	p.F16S	0.20	Yes	Yes	132.6	198.9	40.95	No	2

NYS148	M	7	c.660delC	p.I220IfsX12	0.20	Yes	Yes	140.4	152.1	39.00	No	Novel
NYS412	F	8	c.910C>T	p.R304RX	0.12	Yes	Yes	126.8	195.0	42.90	No	4
NYS605	M	43	c.796G>C	p.A266P	0.18	Yes	Yes	109.2	173.6	39.00	No	1, 2
NYS875	M	35	c.796G>C	p.A266P	0.18	Yes	Yes	148.2	179.4	48.75	No	1, 2
NYS1324	M	58	c.673T>C	p.W225R	0.24	Yes	Yes	138.5	191.1	42.90	No	Novel
NYS175	F	24	c.673T>C	p.W225R	0.10	Yes	Yes	150.2	179.4	48.75	No	Novel
NYS772	M	79	c.206-5T>A		0.34	Yes	Yes	81.9	214.5	37.05	Yes	Novel
NYS301	F	38	c.206-5T>A		0.30	Yes	Yes	66.3	251.6	40.95	Yes	Novel
NYS423	M	25	c.782G>A	p.R261Q	0.16	Yes	Yes	124.8	183.3	46.80	No	4
NYS1040	F	30	c.782G>A	p.R261Q	0.28	Yes	Yes	122.9	185.3	44.85	No	4
NYS1066	F	28	c.824A>G	p.H275R	0.20	Yes	Yes	148.2	195.0	44.85	No	Novel
NYS1182	F	41	c.824A>G	p.H275R	0.28	Yes	Yes	126.8	202.8	46.80	No	Novel
NYS7	M	50	c.285-118C>T		0.30	Yes	Yes	105.3	230.1	40.95	Yes	Novel
NYS134	M	62	c.154G>T	p.G52X	0.18	Yes	Yes	132.6	198.9	39.00	No	Novel
NYS207	M	27	c.811T>A	p.C271S	0.00	Yes	Yes	142.4	181.4	39.00	No	2
NYS1086	M	24	c.285-118C>T		0.38	Yes	Yes	52.7	257.4	37.05	Yes	Novel
NYS1211	M	12	c.285-118C>T		0.30	Yes	Yes	56.6	237.9	40.95	Yes	Novel

References:

1. Tarpey P., Thomas S., Sarvananthan N., Mallya U., Lisgo S., Talbot C.J., Roberts E.O., Awan M., Surendran M., McLean R.J. *et al.* (2006) Mutations in FRMD7, a newly identified member of the FERM family, cause X-linked idiopathic congenital nystagmus. *Nat. Genet.,* **38**, 1242-1244.

2. Thomas M.G., Crosier M., Lindsay S., Kumar A., Thomas S., Araki M., Talbot C.J., McLean R.J., Surendran M., Taylor K. *et al.* (2011) The clinical and molecular genetic features of idiopathic infantile periodic alternating nystagmus. *Brain,* **134**, 892-902.

3. Zhang Q., Xiao X., Li S. and Guo X. (2007) FRMD7 mutations in chinese families with X-linked congenital motor nystagmus. *Mol. Vis.,* **13**, 1375-1378.

4. Li N., Wang L., Cui L., Zhang L., Dai S., Li H., Chen X., Zhu L., Hejtmancik J.F. and Zhao K. (2008) Five novel mutations of the FRMD7 gene in chinese families with X-linked infantile nystagmus. *Mol. Vis.,* **14**, 733-738.
