# Supplementary material for: Abnormal retinal development associated with FRMD7 mutations
Source: Hum Mol Genet. 2014 Mar 31;23(15):4086–93. doi: 10.1093/hmg/ddu122 (PMC4082370; doi:10.1093/hmg/ddu122)

**Supplementary figure 1:** Example of the normal retinal laminar structure visualised using optical coherence tomography. The tomogram represents a B-scan through the foveal pit. The fovea is a specialised structure consisting of a deep foveal pit (a), absence of inner retinal layers posterior to the foveola (b) and lengthening of the outer segment of the cone photoreceptors (c). NFL = nerve fibre layer, GCL = ganglion cell layer, IPL = inner plexiform layer, INL = inner nuclear layer, OPL = outer plexiform layer, ONL = outer nuclear layer, ISe = inner segment ellipsoid, OS = outer segment and RPE = retinal pigment epithelium.


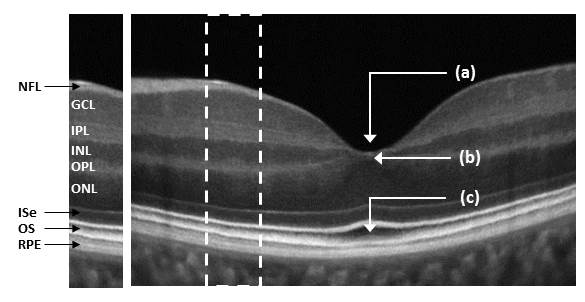

Supplement: Supplementary Data [file supp_ddu122_ddu122supp_fig1.doc]
