# Supplementary material for: Abnormal retinal development associated with FRMD7 mutations
Source: Hum Mol Genet. 2014 Mar 31;23(15):4086–93. doi: 10.1093/hmg/ddu122 (PMC4082370; doi:10.1093/hmg/ddu122)

**Supplementary Table 1**

Forward and reverse primer sequences, product sizes and melting temperature (Tm) used to

amplify *FRMD7* exons.


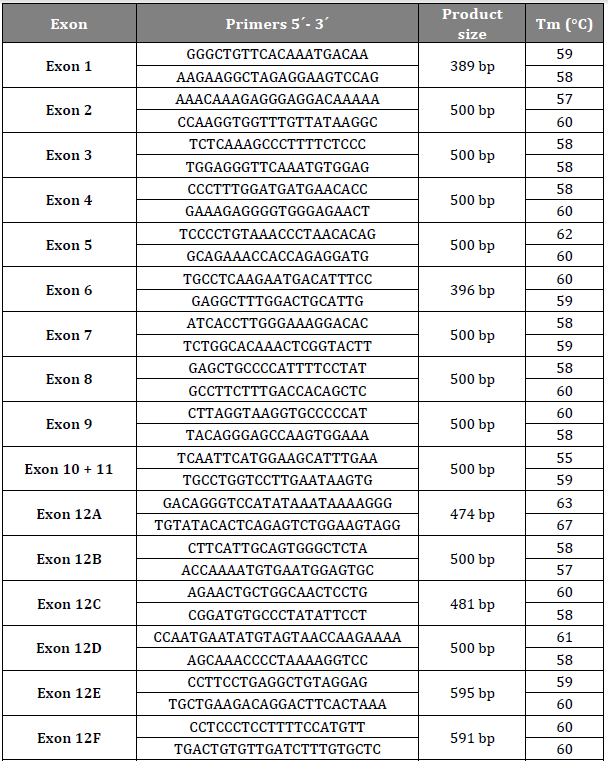

Supplement: Supplementary Data [file supp_ddu122_ddu122supp_table1.docx]
